# Prediction of response to anti-EGFR antibody-based therapies by multigene sequencing in colorectal cancer patients

**DOI:** 10.1186/s12885-015-1752-5

**Published:** 2015-10-27

**Authors:** Laura Lupini, Cristian Bassi, Jitka Mlcochova, Gentian Musa, Marta Russo, Petra Vychytilova-Faltejskova, Marek Svoboda, Silvia Sabbioni, Radim Nemecek, Ondrej Slaby, Massimo Negrini

**Affiliations:** 1 Department of Morphology, Surgery and Experimental Medicine, University of Ferrara, Via Luigi Borsari 46, 44121 Ferrara, Italy; 2 Central European Institute of Technology (CEITEC), Molecular Oncology II, University Campus Bohunice Building A3, Kamenice 5, 625 00 Brno, Czech Republic; 3 Department of Comprehensive Cancer Care, Masaryk Memorial Cancer Institute, Brno, Czech Republic; 4 Department of Life Sciences and Biotechnology, University of Ferrara, Via Luigi Borsari 46, 44121 Ferrara, Italy

**Keywords:** Colorectal cancer, Resistance to anti-EGFR antibodies, Gene mutations, Next-generation sequencing

## Abstract

**Background:**

The anti-epidermal growth factor receptor (EGFR) monoclonal antibodies (moAbs) cetuximab or panitumumab are administered to colorectal cancer (CRC) patients who harbor wild-type *RAS* proto-oncogenes. However, a percentage of patients do not respond to this treatment. In addition to mutations in the *RAS* genes, mutations in other genes, such as *BRAF*, *PI3KCA*, or *PTEN*, could be involved in the resistance to anti-EGFR moAb therapy.

**Methods:**

In order to develop a comprehensive approach for the detection of mutations and to eventually identify other genes responsible for resistance to anti-EGFR moAbs, we investigated a panel of 21 genes by parallel sequencing on the Ion Torrent Personal Genome Machine platform. We sequenced 65 CRCs that were treated with cetuximab or panitumumab. Among these, 37 samples were responsive and 28 were resistant.

**Results:**

We confirmed that mutations in EGFR-pathway genes (*KRAS*, *NRAS*, *BRAF*, *PI3KCA*) were relevant for conferring resistance to therapy and could predict response (*p* = 0.001). After exclusion of *KRAS*, *NRAS*, *BRAF and PI3KCA* combined mutations could still significantly associate to resistant phenotype (*p* = 0.045, by Fisher exact test). In addition, mutations in *FBXW7* and *SMAD4* were prevalent in cases that were non-responsive to anti-EGFR moAb. After we combined the mutations of all genes (excluding *KRAS*), the ability to predict response to therapy improved significantly (*p* = 0.002, by Fisher exact test).

**Conclusions:**

The combination of mutations at *KRAS* and at the five gene panel demonstrates the usefulness and feasibility of multigene sequencing to assess response to anti-EGFR moAbs. The application of parallel sequencing technology in clinical practice, in addition to its innate ability to simultaneously examine the genetic status of several cancer genes, proved to be more accurate and sensitive than the presently in use traditional approaches.

**Electronic supplementary material:**

The online version of this article (doi:10.1186/s12885-015-1752-5) contains supplementary material, which is available to authorized users.

## Background

Colorectal cancer (CRC) is the third most frequent neoplasm worldwide and the third most common cause of cancer-related death in Western countries. CRC is treated with surgical resection and/or systemic chemotherapy based on fluorouracil, irinotecan, oxaliplatin, or capecitabine. Cetuximab and panitumumab, monoclonal antibodies (moAbs) that target the epidermal growth factor receptor (EGFR), are also used in metastatic CRC. However, only 10–20 % of patients affected by metastatic CRC are responsive to this treatment [1].

EGFR protein promotes cell growth and survival signaling through the activation of MAPK and PI3K pathways. Mutation analysis of genes belonging to these pathways revealed that alterations in *KRAS*, as well as in *N*- and *HRAS*, represented biomarkers of the lack of response to cetuximab [1, 2]. As a result, *RAS* mutation analysis was introduced into clinical guidelines for the selection of patients amenable to cetuximab treatment. The focused use of cetuximab against tumors harboring wild-type *RAS* improved its overall usefulness. However, 35–45 % of wild-type *RAS* cases still do not respond to this treatment. Additional studies have now indicated that other elements of the MAPK and PI3K pathways, such as *BRAF*, *PI3KCA*, or *PTEN*, may be involved [1, 3–5]. These findings led to updated guidelines for CRC treatments, which advocated the inclusion of the mutational status of both *KRAS* and *NRAS* genes and the consideration of *BRAF* mutations in wild-type *RAS* cancers [6]. High-throughput sequencing methods, thanks to their ability to analyze several genes in parallel, could represent a helpful support in detecting the numerous genetic changes implicated in anti-EGFR moAb resistance.

With massive parallel sequencing, millions of fragments of DNA can be sequenced in the same reaction, allowing the acquisition of in-depth information that traditional Sanger sequencing cannot readily achieve. For this reason, the use of parallel sequencing technologies is rapidly expanding. In addition to instruments that can sequence full human genomes, “bench” sequencers with lower throughput—but reduced running costs and faster turnaround time—are becoming common. These bench sequencing systems are more apt when a relatively small number of genes need to be sequenced. Sample preparation and data analysis are compatible with barcoding, meaning that multiple samples can be labeled and loaded in the same sequencing assay, allowing consistent time and cost savings. For these reasons, in addition to simpler data analysis, this type of sequencer can be more easily accommodated in a clinical setting.

In this study, we selected a group of 21 genes involved in CRC [1, 7] to sequence 65 CRCs from patients treated with cetuximab or panitumumab by using the Ion Torrent Personal Genome Machine (PGM) platform. The study proved the usefulness of parallel sequencing, confirmed earlier reports about the genes involved in cetuximab resistance, and revealed a potential important role for *FBXW7* and *SMAD4* mutations in conferring therapy resistance to anti-EGFR moAbs.

## Methods

### Clinical samples

Samples were obtained from 65 patients with histologically confirmed colorectal adenocarcinoma and undergoing surgery at the Masaryk Memorial Cancer Institute (MMCI, Brno, Czech Republic) between 2004 and 2011. Patient age ranged from 31 to 81 years with a mean of 58 years. The Ethical committee of the Masaryk Memorial Cancer Institute approved the study protocol. Written informed consent was obtained from all patients. All participants included in the study were anonymized by using sample identifiers that could not be connected with any individual. Clinicopathological features of the patients are summarized in Table 1 and Additional file 1: Figure S1. At the time the samples were collected, the TheraScreen K-RAS Mutation Kit CE-IVD was in use. The test allowed analysis of the mutational status at codons 12 and 13 of *KRAS* only. According to the results of this test, all 65 samples carried wild-type *KRAS*, and patients were treated with cetuximab or panitumumab. At the time of first diagnosis, tumors of some patients were at stages I-III, but at the time of anti-EGFR moAb treatment, all patients were at stage IV. Patients were regularly followed up after beginning this treatment. End points of follow-up were death and progression of disease. Cetuximab response was assessed according to RECIST (Response Evaluation Criteria In Solid Tumors) criteria. Enrolled patients were divided into two groups: one group (responders) included patients with a complete response (CR; 100 % reduction of metastasis) or a partial response (PR; >30 % reduction of metastasis) or with stabilization of the disease (SD), whereas a second group (non-responders) included patients with progressive disease (PD).

**Table 1:** Clinical features of colon cancer samples

		Non-responders	Responders
Samples		28 (PD)	37
			7 (CR)
			19 (PR)
			11 (SD)
Age, years		58.3	57.9
Gender	M	18	24
	F	10	13
Localization	R	6	1
	L	21	34
	ND	1	2
Stage at first diagnosis	I	2	0
	II	1	5
	III	5	2
	IV	20	29
	ND	0	1
Grade	G1	2	3
	G2	24	30
	G3	1	1
	ND	1	3
Treatment	Cetuximab	23	29
	Panitumumab	5	8
OS, average months		34.8	61.5
TTP, average months		3.6	14.9

*PD* progressive disease, *CR* complete response, *PR* partial response, *SD* stable disease, *M* male, *F* female, *R* right colon, *L* left colon, *OS* overall survival, *TTP* time to progression, *ND* not determine

### Gene selection and primer design

The CRC gene panel was assembled by considering the 19 most frequently mutated genes in non-hypermutated CRCs [7]; to these, *EGFR* and *BRAF* genes were added for their involvement in the EGFR pathway [1, 3–5]. Gene regions and the 584 primer pairs are listed in Additional file 2: Table S1. Primer pairs for the amplification of each gene region of interest were designed by using AmpliSeq Designer v.1.2.6 software [8] (Life Technologies).

### Isolation of DNA and sample selection

DNA was isolated from formalin-fixed, paraffin-embedded (FFPE) samples by using the QIAamp DNA FFPE Tissue Kit (Qiagen) according to the manufacturer’s protocol. No enrichment for tumor cells was done, however, according to histopathological analysis, the average percentage of tumor cells in tissue sections was 70 %. The concentration of DNA was ascertained with the Qubit 2.0 Fluorometer (Life Technologies) by using the Qubit dsDNA HS Assay Kit (Life Technologies).

### Library preparation and sequencing

Library preparation was performed according to the Ion AmpliSeq Library Kit 2.0 protocol (Life Technologies), starting with 20 ng of genomic DNA. Two 20-cycle multiplex amplification reactions of the regions of interest were performed by using AmpliSeq custom oligos. An Ion Xpress Barcode Adapters Kit (Life Technologies) was used to add Ion Torrent specific motifs to amplicons. For purification, an Agencourt AMPure XP reagent (Beckman Coulter) was used. Final libraries were quantified by using the Bioanalyzer instrument with the High Sensitivity DNA Kit (Agilent), diluted and pooled together in equimolar amounts. Twenty microliters of a 16 pM pool of all libraries was mixed with Ion Sphere Particles and clonally amplified in an emulsion PCR, performed in accordance with the Ion OneTouch 200 Template kit v.2 DL protocol and using the Ion OneTouch instrument (Life Technologies). Enrichment-System and Dynabeads MyOne Streptavidin C1 magnetic beads (Life Technologies) were used to enrich template-positive Ion Sphere Particles. Enriched samples were loaded onto Ion 316 (up to 8 samples) or 318 chips (up to 17 samples) and sequenced by using the Ion Torrent PGM, following the Ion PGM 200 Sequencing Kit protocol.

### Data analysis and variant identification

Sequencing data analysis was conducted by using Torrent Suite software v. 3.4 (Life Technologies). Briefly, low-quality reads were removed, adapter sequences trimmed, and alignment against a reference genome (hg19) performed by using the Torrent Mapping Alignment Program. The Variant Caller plugin was used to identify variations from the reference sequence. To identify pathogenic variations, mutations that did not affect the protein coding regions (intronic, 3’ and 5’ UTR variations and silent exonic mutations) were filtered out; insertions and deletions belonging to homopolymeric regions were removed, because sequencing error rate is high in these regions; alterations found at a frequency lower than 15 % were excluded, based on the hypothesis that mutations at lower frequencies could marginally affect tumor behavior and because this filtering step allowed to remove most of variations derived from formalin fixation artifacts [9]. Remaining mutations were compared with data present in the public databases dbSNP [10], COSMIC [11], and cBIO [12] to search for known pathogenic mutations. Annotated non-pathogenic variations were excluded from results, whereas remaining potentially pathogenic variations and mutations of unknown significance were retained. At the same time, Annovar [13] and Mutation Assessor [14] algorithms were used to predict damaging or potentially damaging changes in tumor suppressor genes.

### Statistical analysis

The association of gene mutations with anti-EGFR treatment resistance was evaluated by using a two-tailed Fisher’s exact test. GraphPad Prism 5 was used to perform survival analysis.

### Sanger sequencing

Sanger sequencing was performed according to standard procedure. Amplicons were prepared using the same primer pairs employed for library preparation and were sequenced using the following sequencing primers: CGT CCA CAA AAT GAT TCT GAA TTA GC for amino acids 12 and 13 of *KRAS*; TGC ACT GTA ATA ATC CAG ACT GTG TTT for amino acid 61 of *KRAS*; GGA TTA AGA AGC AAT GCC CTC TCA for amino acid 146 of *KRAS*; GAT TTT TGT GAA TAC TGG GAA CTA TG for amino acids 600 and 616 of *BRAF*; TGT AGA TGT GGC TCG CCA ATT AA for amino acid 12 of *NRAS* and TTG AAC TTC CCT CCC TCC CT for amino acid 61 of *NRAS*; CCT TGA CTA AAT CTA CCA TGT TTT CTC A for amino acids 479, 481, and 505 of *FBXW7*; ATG CCT TCA TTT TTC TCT TCA CCA GTA for amino acid 399 of *FBXW7*; CGT GTG GTA GAG GAG GAA CAG for amino acid 81 of *FBXW7*; and GGT CAG TAA TTG ATA GGA AGA GTA TCC A for amino acid 582 of *FBXW7*.

## Results

### Detection of anti-EGFR treatment-related genes through next-generation sequencing

The DNA of primary tumor lesions from 65 advanced CRCs treated with cetuximab or panitumumab (Table 1, Additional file 1: Figure S1) was investigated. Thirty-seven patients were responsive to therapy, whereas 28 displayed progression of disease. The two groups were balanced for age, sex, stage, and grade. A slight difference in tumor localization was present, with a prevalence of right colon cancers in the non-responder group (*p* = 0.04). All samples were negative for *KRAS* mutations according to the TheraScreen K-RAS Mutation Test.

We investigated the coding sequences of 21 genes, selected according to the previously reported genes most frequently mutated in CRCs [3, 5, 7] or present in public cancer mutations databases, such as COSMIC [11] and cBioPortal [12] (Additional file 2: Table S1). Amplicon libraries and sequencing reactions were performed as described in the Methods.

All designed gene segments (*n* = 584) were sequenced with an average coverage of 506 reads each. Variations were identified in comparison with a human nucleotide reference sequence (hg19) by the Variant Caller plugin (Torrent Suite v. 3.4). To identify potentially pathogenic variations, we filtered out some identified nucleotide changes, as indicated in the Materials and Methods. All of the remaining variations are listed in Additional file 3: Table S2.

**Fig. 1:**
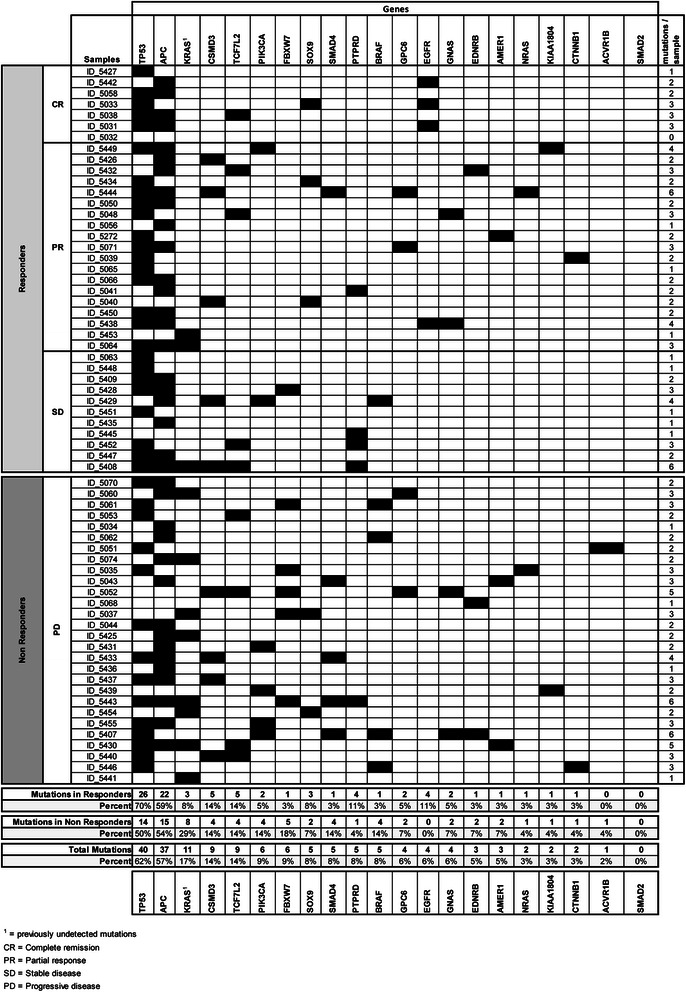
Mutations in the 21-gene panel in tumor samples from patients who underwent anti-EGFR therapy. Black squares indicate the presence of a mutation. The overall frequencies of gene mutations, as well as the frequencies in the responder and non-responder groups, are shown at the bottom of the figure

At least one mutation was detected in each sample, the only exception being sample ID_5032. This sample showed a complete response to anti-EGFR treatment. The most frequently mutated genes were *TP53* and *APC*, which were mutated in 40 (62 %) and 37 samples (57 %), respectively. These results are in agreement with the published literature, which, besides confirming the importance of these two genes for CRC pathogenesis, validates the reliability of our sequencing results. The mutation rate for the remaining genes was as follows: 17 % for *KRAS* (additional mutations, previously undetected by TheraScreen test), 14 % for *CSMD3*, 14 % for *TCF7L2*, 9 % for *PIK3CA* and *FBXW7*, and less for the other genes. No mutation was detected in *SMAD2* (Fig. 1).

Mutation rates for each gene in the responder and non-responder groups are shown in Fig. 1. The two genes with the highest mutation frequency, *TP53* and *APC*, displayed mutations in both groups: *TP53* had a higher mutation frequency in responders than in non-responders (70 % versus 50 %), but the difference was not significant (*p* = 0.125); *APC* showed a similar mutation frequency in both responders and non-responders (59 % versus 54 %).

Despite initial analyses based on TheraScreen KRAS Mutation Test indicating that all tumors carried a wild-type *KRAS* gene, the subsequent next-generation sequencing (NGS) analysis revealed that 11 primary tumors harbored a mutation in this gene. In particular, we found the following variations: G12C, G12V, G13D, Q61H, Q61L, and A146T. All of these alterations were previously described as pathogenic for the *KRAS* gene and recorded in the COSMIC database. Eight of the 11 samples were found in patients who were resistant to anti-EGFR treatment, whereas the other three *KRAS* mutated samples belonged to the responder group. Of these three responsive tumors, one sample with a Q61H variation (detected in 54 % of reads) was from a patient with stable disease, and two samples harboring Q61H and G13D (detected in less than 18 % of reads) were from patients who showed a partial response to treatment. These additional, previously undetected mutations in the KRAS gene alone were significantly associated with the appearance of drug resistance (*p* = 0.045) (Table 2 and Additional file 4: Table S3).

**Table 2:** Correlation between mutational status and resistance to anti-EGFR moAb therapy

Mutant genes	Responders (CR + PR + SD)^a^ vs non-responders				
	Total responders	Total non-responders	Mutant responders	Mutant non-responders	*P*-value^b^
*KRAS* ^c^	37	28	3	8	0.045
*NRAS*	37	28	1	1	0.999
*BRAF*	37	28	1	4	0.156
*PI3KCA*	37	28	2	4	0.389
*FBXW7*	37	28	1	5	0.077
*SMAD4*	37	28	1	4	0.156
*NRAS*/*BRAF*/*PIK3CA* ^d^	37	28	3	8	0.045
*NRAS*/*BRAF*/*PIK3CA*/*FBXW7* ^d^	37	28	4	11	0.016
*NRAS*/*BRAF*/*PIK3CA*/*FBXW7*/*SMAD4* ^d^	37	28	4	13	0.002

^a^ *CR* complete response, *PR* partial response, *SD* stable disease

^b^ *P*-value from Fisher's exact test

^c^Previously undetected mutations

^d^Samples with mutations in at least one of the indicated genes

Besides *KRAS*, the mutational status of *BRAF*, *NRAS*, and *PIK3CA* was already shown to correlate with cetuximab resistance. In this study, *BRAF* and *PI3KCA* genes displayed an imbalance (>2 fold), albeit not a statistically significant one, in mutations detected in the responder patients as compared with those in the non-responder patients (Table 2). We detected five *BRAF* mutations (V600E and S616F), two *NRAS* (Q61R and G12D), and six *PIK3CA* variations (R38H, I391M, and H1047L). All of these variations were annotated in the COSMIC database. Considering a combination of these three genes, eight mutations belonged to tumors that did not respond to therapy, and three mutations were in samples from patients that showed either a partial response or stable disease (Additional file 3: Table S2). Notably, mutations in *KRAS*, *BRAF*, *NRAS*, and *PIK3CA* appeared to be mutually exclusive (Fig. 1 and Additional file 1: Figure S2). Because these four genes are downstream effectors of the EGFR-induced pathways, they appear to be functionally significant in driving cetuximab or panitumumab resistance. Combined *NRAS*, *BRAF* and *PIK3CA* mutation frequency significantly correlated to anti-EGFR resistance phenotype (*p* = 0.045) (Table 2). If additional *KRAS* mutations, found by NGS, were considered in addition to the three genes of the panel, the combined mutation frequency became highly significantly correlated with resistance to anti-EGFR therapy (*p* = 0.001) (Additional file 4: Table S3).

**Fig. 2:**
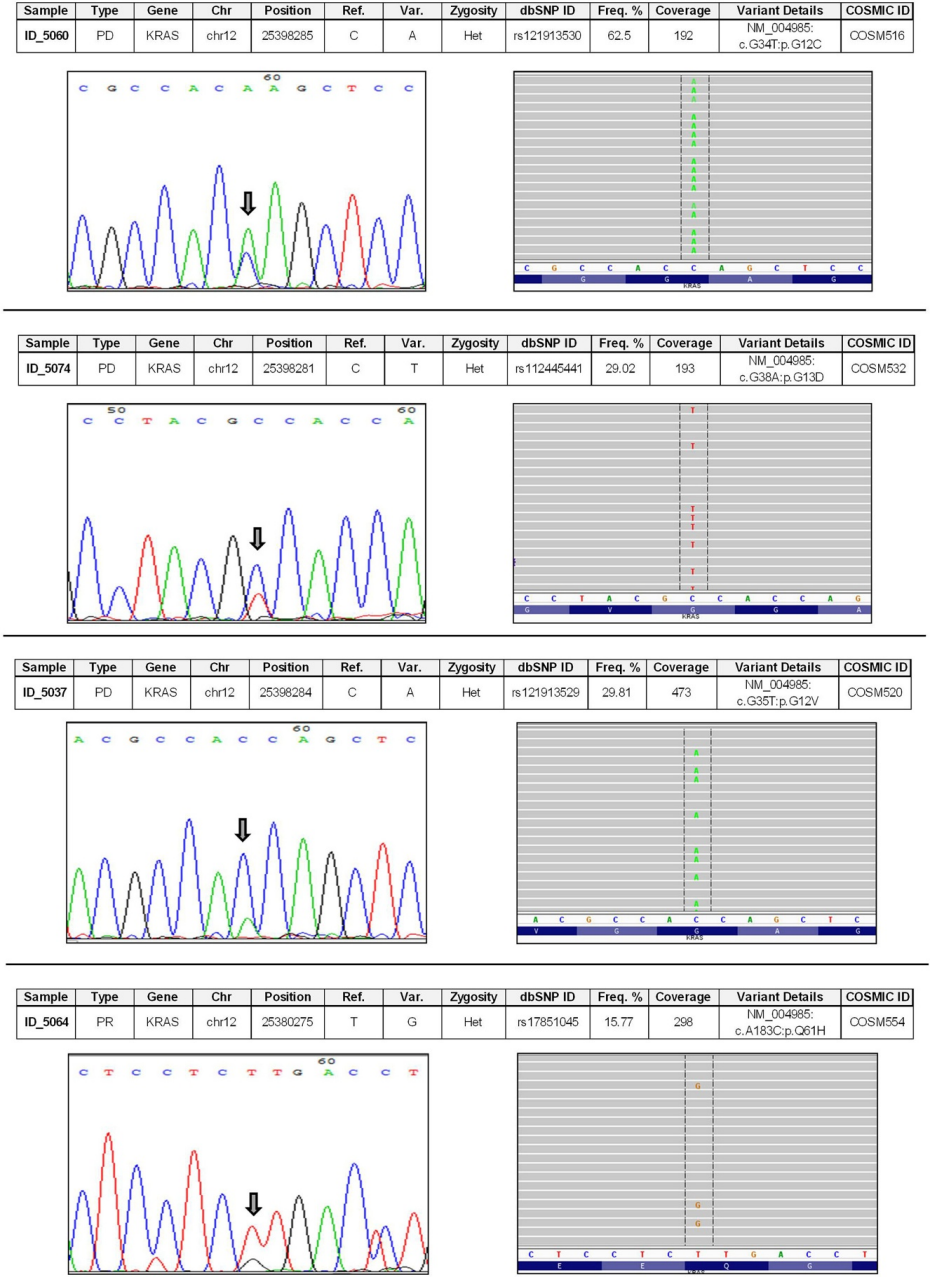
Sanger sequencing validation of some *KRAS* mutations. All 11 *KRAS* mutations identified by NGS were also validated by using Sanger sequencing. The figure shows a comparison between NGS and Sanger sequencing results for four *KRAS* mutations. On the left are displayed Sanger chromatograms, while results of NGS, showing read alignment to the reference genome, are on the right of the panel

To validate these findings, we confirmed the presence of mutations in *KRAS*, *BRAF*, and *NRAS* genes by the standard Sanger sequencing method (Fig. 2 and Additional file 1: Figure S3). From the chromatograms, we noticed that mutant nucleotides were called by Sanger sequencing only in the case of high-frequency mutations (e.g., *KRAS* G12C, found in 63 % of reads of sample ID_5060), whereas in the vast majority of samples, mutant nucleotides could be observed by visual inspection of chromatograms, but they were not called by the analysis software, as wild-type nucleotides were prevalent. These results confirm the better qualitative and quantitative accuracy of NGS data.

**Fig. 3:**
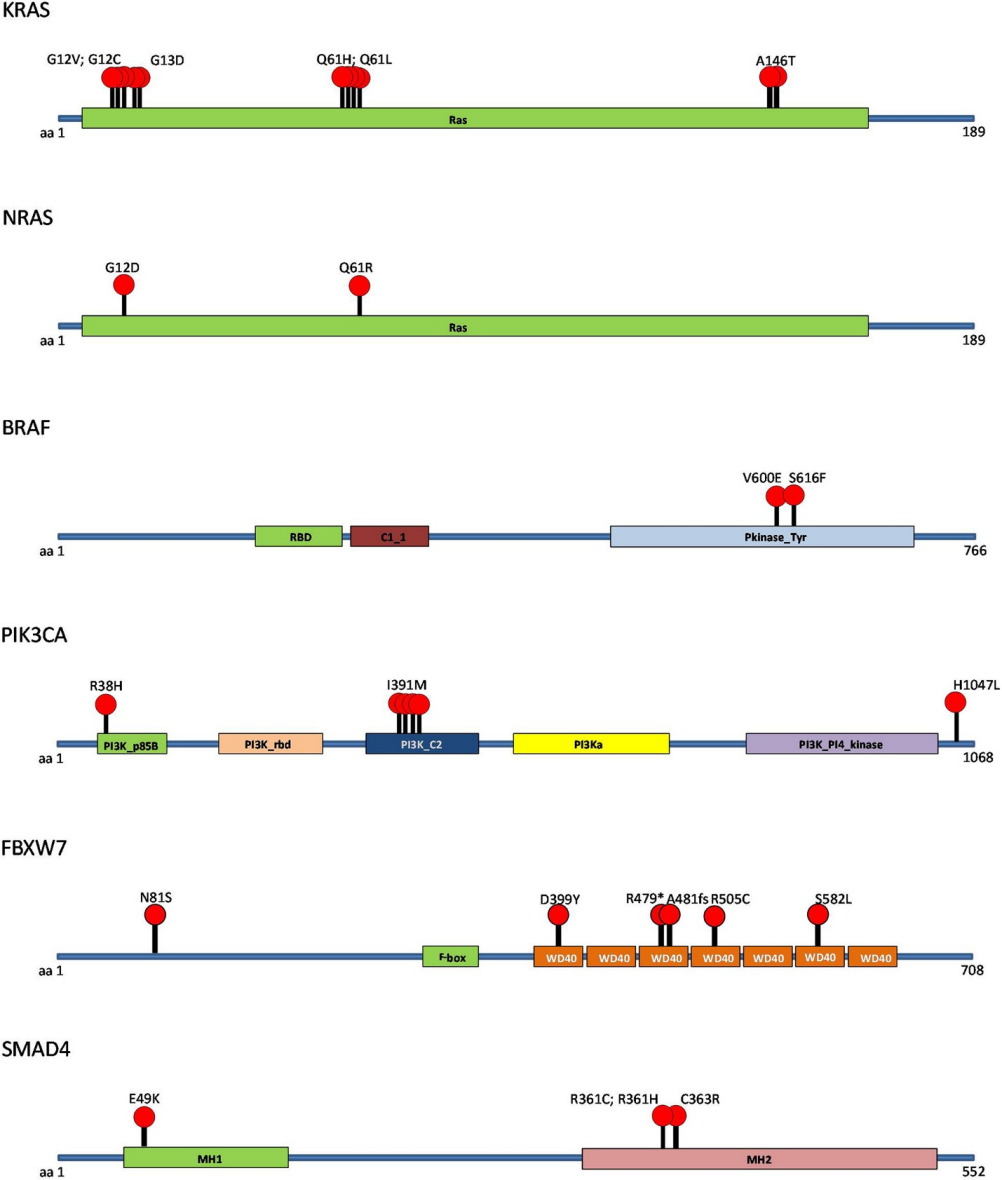
Gene mutation diagram. Position of mutations found in KRAS, NRAS, BRAF, PIK3CA, FBXW7, and SMAD4 proteins. Red circles indicate the position of mutations within protein domains, which are responsible for protein-protein interactions or enzymatic activities. RBD, Raf-like Ras-binding domain; C1_1, phorbol esters/diacylglycerol binding domain (C1 domain); Pkinase_Tyr, protein tyrosine kinase; PI3K_p85B, PI3-kinase family, p85-binding domain; PI3K_rbd, PI3-kinase family, ras-binding domain; PI3K_C2, phosphoinositide 3-kinase C2; PI3Ka, phosphoinositide 3-kinase family, accessory domain (PIK domain); PI3_PI4_kinase, phosphatidylinositol 3- and 4-kinase; WD40, WD domain, G-beta repeat

Besides genes of the EGFR pathway, the other genes most frequently mutated in CRCs included *CSMD3* (14 %), *TCF7L2* (14 %), and *FBXW7* (9 %) (Fig. 1). Whereas *CSMD3* and *TCF7L2* mutations were equally distributed between the two groups, mutations in *FBXW7* were prevalent (6.6-fold difference) in resistant samples, as they were found in five resistant samples and in only one responder belonging to the stable disease subgroup (*p* = 0.077) (Table 2). The *FBXW7* mutations included a nucleotide insertion involving amino acid 481 (A481fs) in 15 % of reads; a nonsense mutation R479* (COSM206697) in 57 % of reads; two missense variations (D399Y and N81S) in 19 and 57 % of reads, respectively; the variation R505C in 24 % of reads (COSM22975); and missense mutation S582L (COSM22979) found in 21 % of reads. With the exception of N81S, which is a variation of unknown clinical significance annotated in the dbSNP database (rs139738471), all of the other variations occur inside WD-40 domains (also known as WD or beta-transducin repeats) (Fig. 3), which are responsible for protein-protein interactions. These five mutations are predicted to have significant effects on protein stability, suggesting that they interfere with the production of a functional FBXW7 protein. As mentioned earlier, the only responder sample with a *FBXW7* mutation (ID_5428 with mutation S582L in 21 % of reads) belonged to the stable disease group. If samples from the borderline stable disease group are not considered, the statistical association between *FBXW7* mutations and resistance to cetuximab becomes significant (*p* = 0.05). Moreover, if the *FBXW7* mutations are considered together with *BRAF*, *NRAS*, and *PIK3CA* alterations, the significance of the association with the chemo-response is further increased (*p* = 0.016) (Table 2). All of the mutations in *FBXW7* were further validated by Sanger sequencing (Additional file 1: Figure S3). Mutations in the *SMAD4* gene also exhibited an imbalance (5.3-fold change) between the non-responder and the responder patients: four mutations were found in samples from the non-responders and one mutation was found in a patient with a partial response to anti-EGFR therapy (Fig. 1 and Additional file 1: Figure S2). As in all of the other individual genes, with the exception of *KRAS*, the difference was not statistically significant (*p* = 0.16) (Table 2). However, if all of the genes with imbalanced mutations (excluding *KRAS*) are considered in responders versus non-responders, 13 cases (46 %) exhibit a mutation in at least one of the five genes (*NRAS*, *BRAF*, *PI3KCA*, *FBXW7*, and *SMAD4*) among the non-responders and 4 (11 %) among the responders. This difference is statistically highly significant (*p* = 0.002) (Table 2). If the additional *KRAS* variations are included within the five gene panels, significance strikingly improves (*p* = 0.0001) (Additional file 4: Table S3).

## Discussion

Anti-EGFR therapy based on cetuximab or panitumumab moAbs is administered to treat advanced CRCs that carry a wild-type *KRAS* gene [1, 3–5]. Nonetheless, some patients do not respond to this therapy, as genes other than *KRAS* are involved in the resistance to anti-EGFR molecules. Indeed, previous work has reported the involvement of mutations in genes such as *BRAF*, *PIK3CA*, and *PTEN* [1, 3–5]. All of these genes represent down-stream effectors or modulators of the EGFR pathway, thus establishing a rationale for the anti-EGFR moAb response. More recently, new guidelines for the use of cetuximab and panitumumab treatment in CRC patients supported the analysis of the mutational status of both *KRAS* and *NRAS* genes and *BRAF* in wild-type *RAS* cancers [6]. Samples analyzed in this study were antecedent to these guidelines and were evaluated only for the mutational status of *KRAS* (codons 12 and 13).

Here, we performed a targeted resequencing of a group of genes previously reported as the most frequently mutated genes in non-hypermutated CRCs [7]: *TP53*, *APC*, *KRAS*, *CSMD3*, *TCF7L2*, *PI3KCA*, *FBXW7*, *SOX9*, *SMAD4*, *PTPRD*, *GPC6*, *EDNRB*, *GNAS*, *AMER1*, *NRAS*, *KIAA1804*, *CTNNB1*, *ACVR1B*, and *SMAD2*. Analysis of *EGFR* and *BRAF* were added for their known involvement in the EGFR signaling pathway. Using this 21-gene panel, we investigated 65 CRC samples from patients treated with anti-EGFR moAbs to uncover genes whose mutational status could be associated with differential sensitivity to therapy. This study proves the feasibility of high-throughput sequencing of several genes in large numbers of samples to get detailed information about the mutational status of analyzed genes and it highlights the better sensitivity of NGS technologies compared to traditional capillary sequencing.

The most frequently mutated genes in our samples were *TP53* and *APC*. Since these two genes have been previously described as the most frequently mutated in CRC [7, 15], this finding validates the reliability of our sequencing results. Although a higher percentage of mutant *TP53* was detected in responders, no significant correlation between the mutational status of these two genes and the resistance phenotype was found (*p* = 0.125). We also found that a number of genes, including *KRAS* (previously undetected mutations), *BRAF*, *PI3KCA*, *FBXW7*, and *SMAD4*, exhibited a higher frequency (>2-fold) of mutations in non-responders than in responders.

Notably, although the samples were classified as wild-type *KRAS* by the TheraScreen KRAS Mutation Test, sequencing of primary lesions identified the presence of additional mutations in the *KRAS* gene in 11 samples. This discrepancy partially occurred because the Therascreen test consists of an allele-specific PCR able to detect seven mutations at codons 12 and 13 of the *KRAS* gene, whereas codons 61 and 146, whose clinical significance has now been proven, were not covered by the assay. However, five of the *KRAS* mutations were at codons 12 and 13. A similar underestimation of *KRAS* mutated samples using this assay was previously found by Dono and colleagues [16], and differences between NGS and routine clinical assays have been previously described [17].

Surprisingly, mutations in *KRAS* were also discovered in samples belonging to the responder group. Contrasting significance had already been reported for the *KRAS*-G13D mutation, found in sample ID_5443 [18]. Our results do not help to clarify the issue, since another G13D mutation was detected in a non-responsive patient (ID_5074). Contrasting data were also obtained for the Q61H mutation: two cases were found within the responders (ID_5064 and ID_5408) and one within the non-responders (ID_5454). An additional different mutation at codon 61 (Q61L in sample ID_5430) was found in non-responders. A Q61R mutation was also found to affect *NRAS* in a non-responder case (ID_5035). These findings suggest that a complex picture emerges in deciphering the role of the *RAS* mutation in conferring resistance to moAbs against EGFR; in particular, the response of mutations at codons 13 or 61 may depend on the type of mutation and possibly on other tumor alterations that may affect individual susceptibility.

With the exception of *NRAS*, alterations in genes involved in the EGFR pathway other than *KRAS* generally exhibited an imbalance, albeit at lower frequencies, between responder and non-responder patients. Overall, mutations in *NRAS* were found in 3 % of samples, in *BRAF* in 8 %, and in *PIK3CA* in 9 %. The detected mutation rates overlapped those reported by Smith and colleagues in a study performed on a large series of CRCs [19]. Our data confirmed that mutations in these genes were generally mutually exclusive. The combined mutations in *NRAS*, *BRAF*, and *PIK3CA* could predict resistance to anti-EGFR moAbs with a statistically significant value, as recently also highlighted by Ciardiello and colleagues [17]. The association of mutations in these three genes with the resistant phenotype had a *p*-value of 0.045. Although the newly identified *KRAS* mutations alone significantly associated with anti-EGFR resistant phenotype (*p* = 0.045), we excluded *KRAS* gene from correlation calculations because patients’ cohort was initially selected for wt *KRAS* status, thus potentially producing a bias in *KRAS* mutation frequency. It should be highlighted that, if *KRAS* gene is added to the panel, prediction significance will strikingly increase (Additional file 4: Table S3)”.

Two other genes, the E3 ubiquitin protein ligase F-box and WD repeat domain containing 7 (*FBXW7* or *FBW7*) and the SMAD family member 4 (*SMAD4*), exhibited an imbalance in mutations between responders and non-responders. Mutations in *FBXW7* were identified in six samples (9 %): five in the non-responders and one in the stable disease subgroup of the responders. No *FBXW7* mutation was detected in patients with a partial or a complete response. *FBXW7* mutations alone were associated with a resistant phenotype with a *p*-value of 0.077. However, by adding *FBXW7* to the *NRAS*-*BRAF*-*PIK3CA* mutation panel, the significance of the panel became stronger (*p*-value of 0.016). The involvement of *FBXW7* in resistance to traditional chemotherapies has been previously reported [20] (for a review, see [21]). Mutations in *FBXW7* in human CRCs were previously found in 11–12 % of cases [7, 15] and its low expression was shown to be correlated with a poor prognosis [22]. Down-regulation of *FBXW7* expression was also reported in other cancers [23, 24] and leukemias [25–27]. F-box proteins constitute one of the subunits of the ubiquitin protein ligase complex and they function in the ubiquitin-mediated degradation of several cellular proteins. Genes belonging to the ubiquitin proteasome complex are often mutated in cancer, leading to reduced oncoprotein turnover [28]. In particular, FBXW7 is the component for substrate recognition [21] and, through its F-box domain, is able to bind and mediate the degradation of some known oncogenes, including cyclin E [29], c-Myc [30], c-Jun [31], c-Myb [32], Notch [31], and mTOR [33]. Since nearly all of the mutations we found in *FBXW7* affected the WD-40 domains, which are responsible for protein-protein interactions, and they all appear to be inactivating mutations that are able to interfere with the production of a functional FBXW7 protein, the results of this work provide evidence for a potential role of inactivating mutations in conferring resistance to anti-EGFR moAbs. Importantly, previous mutational studies and animal models have shown that even monoallelic mutations in *FBXW7* could be sufficient to promote tumorigenesis [34, 35], suggesting that these mutations could dominantly affect functionality of the ubiquitin proteasome complex. The mechanism through which mutations in *FBXW7* could impair cetuximab or panitumumab efficacy requires further studies. It is possible that, since some of its proven targets are downstream effectors of EGFR, mutations of *FBXW7* could impair their degradation and thus contribute to the resistant phenotype.

One other gene that appeared potentially interesting is *SMAD4*. Like the mutations in *FBXW7*, mutations in *SMAD4* appear to be unbalanced, with one mutation in a responder sample versus four mutations in resistant samples. Although not significant in itself (*p*-value of 0.16) in our sample cohort, combining mutations in *SMAD4* with those in *NRAS*-*BRAF*-*PIK3CA*-*FBXW7* further improved the significance of the panel (*p*-value of 0.002). Given the function of SMAD4 protein, a possible involvement of a non-functional TGFβ pathway in conferring resistance to anti-EGFR moAbs might be suggested.

## Conclusions

By sequencing a panel of 21 genes involved in CRC, we found that, beside *KRAS* gene, whose previously undetected mutations reached statistical significance as individual gene, the combined mutations of other genes belonging to the EGFR pathway (*NRAS*, *BRAF* and *PIK3CA*) together with mutations at *FBXW7* and *SMAD4* genes achieved a significant association with resistance to anti-EGFR therapy. These results indicate that mutations at *KRAS* combined with the 5 gene panel found in this study, have a strong potential for predicting response to anti-EGFR moAbs. This work supports the usefulness of NGS technology and multigene sequencing over the traditional capillary sequencing for improving patient management in a clinical setting.

### Availability of supporting data

Sequencing data supporting the results of this article are available in the ArrayExpress database [36], with the following accession number: E-MTAB-3883; hyperlink to dataset: http://www.ebi.ac.uk/arrayexpress/experiments/E-MTAB-3883.

## Additional files

Additional file 1: Figure S1. Kaplan Meier plots for progression-free survival (PFS) in the two cohorts (responders and non-responders) of CRC patients. **Figure S2. **Frequencies of mutation in the six genes correlated with anti-EGFR moAb responsiveness. **Figure S3.** Sanger sequencing validation of *BRAF*, *NRAS*, and *FBXW7* mutations. (PDF 668 kb)
Additional file 2: Table S1. Sequences and positions in the human genome of primers employed for amplification of regions of interest. (XLSX 69 kb)
Additional file 3: Table S2. Genetic changes found in all CRC samples. (XLSX 34 kb)
Additional file 4: Table S3. Correlation between mutational status, including KRAS, and resistance to anti-EGFR moAb therapy. (XLSX 12 kb)

## Abbreviations

moAb: Monoclonal antibody
CRC: Colorectal cancer
PGM: Personal genome machine
NGS: Next-generation sequencing
EGFR: Epidermal growth factor receptor
FFPE: Formalin-fixed, paraffin-embedded
