# Supplementary material for: Prediction of response to anti-EGFR antibody-based therapies by multigene sequencing in colorectal cancer patients
Source: BMC Cancer. 2015 Oct 27;15:808. doi: 10.1186/s12885-015-1752-5 (PMC4624582; doi:10.1186/s12885-015-1752-5)
Supplement: Additional file 1: Figure S1. — Kaplan Meier plots for progression-free survival (PFS) in the two cohorts (responders and non-responders) of CRC patients. Figure S2. Frequencies of mutation in the six genes correlated with anti-EGFR moAb responsiveness. Figure S3. Sanger sequencing validation of BRAF, NRAS, and FBXW7 mutations. (PDF 668 kb) [file 12885_2015_1752_MOESM1_ESM.pdf]

ADDITIONAL MATERIAL

SUPPLEMENTARY FIGURES

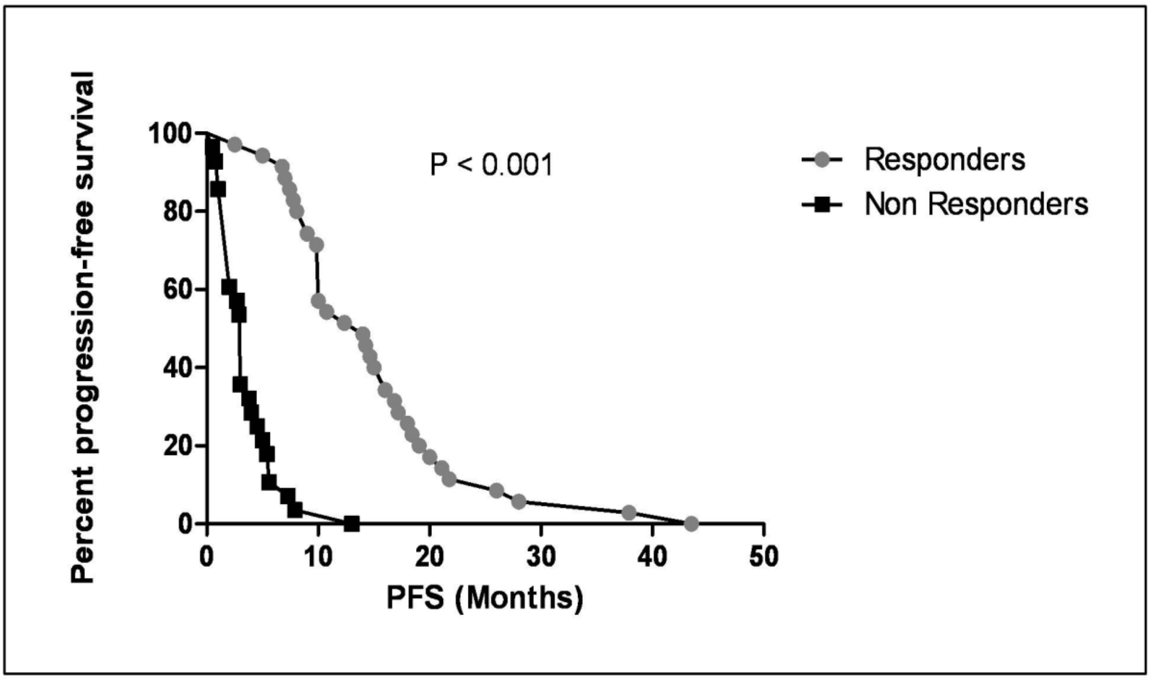

Figure S1. Kaplan Meier plots for progression-free survival (PFS) in the two cohorts (responders and non-responders) of CRC patients. The plots show a significantly different profile in responder versus non-responder patients.

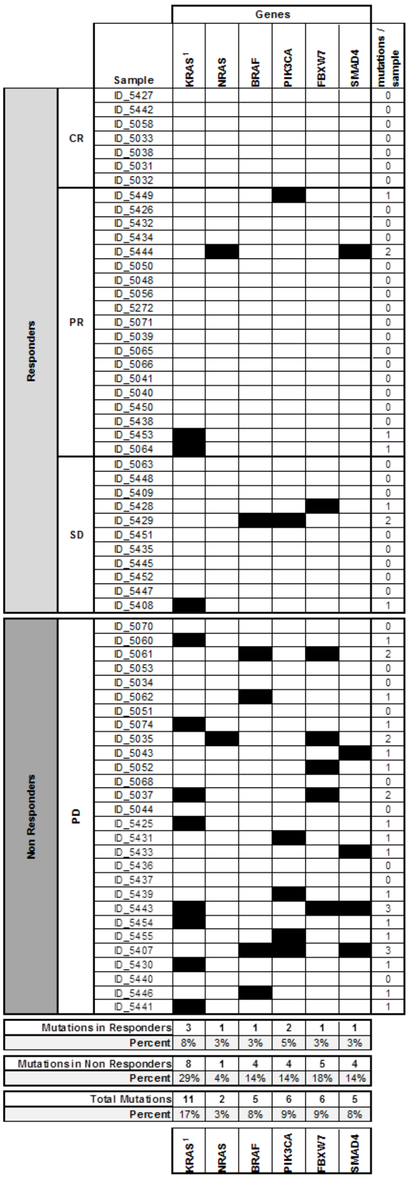

[1] = previously undetected mutations
CR = Complete remission
PR = Partial response
SD = Stable disease
PD = Progressive disease

Figure S2. Frequencies of mutation in the six genes correlated with anti-EGFR moAb responsiveness. The figure shows the mutations detected in genes (*NRAS*, *BRAF*, *PIK3CA*, *FBXW7*, *SMAD4* and previously undetected variations at *KRAS*,) that display imbalanced frequencies in responder versus non-responder patients.

Sample	Type	Gene	Chr	Position	Ref.	Var.	Zygosity	dbSNP ID	Freq. %	Coverage	Variant Details	COSMIC ID
ID_5407	PD	BRAF	chr7	140453136	A	T	Het.	rs113488022	57.2	1984	NM_004333: c.T1799A:p.V600E	COSM476

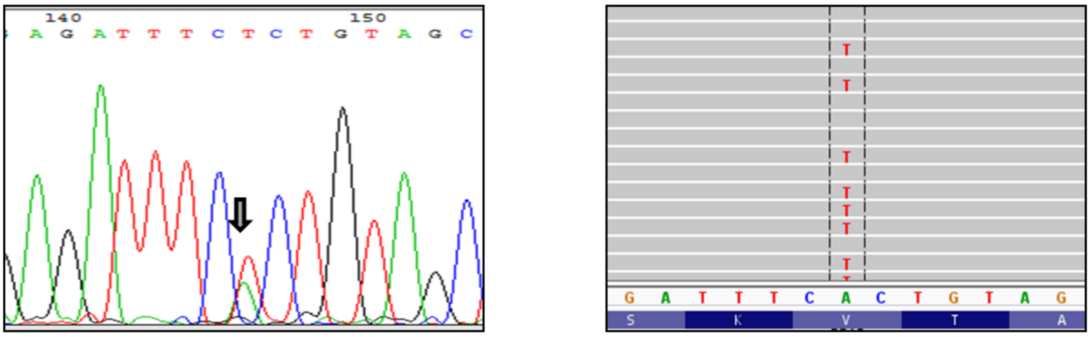

Sample	Type	Gene	Chr	Position	Ref.	Var.	Zygosity	dbSNP ID	Freq. %	Coverage	Variant Details	COSMIC ID
ID_5035	PD	NRAS	chr1	115256529	T	C	Het	rs11554290	50.54	93	NM_002524: c.A182G:p.Q61R	COSM584

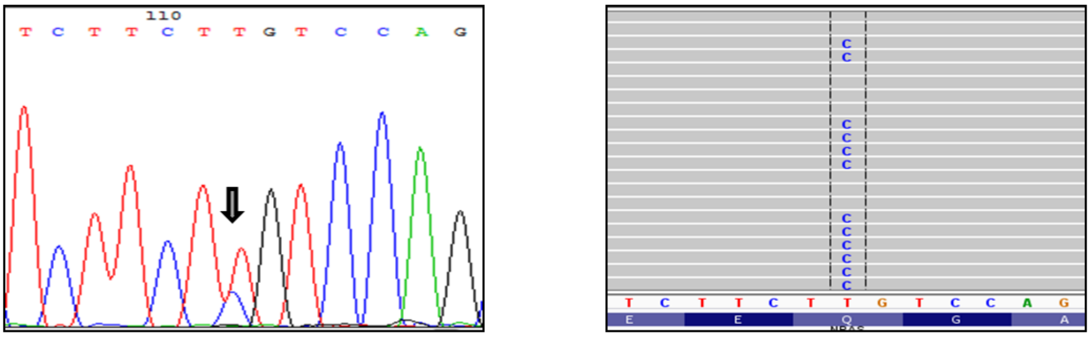

Sample	Type	Gene	Chr	Position	Ref.	Var.	Zygosity	dbSNP ID	Freq. %	Coverage	Variant Details	COSMIC ID
ID_5428	SD	FBXW7	chr4	153245446	G	A	Het.		20.7	314	NM_001013415: c.C1391T:p.S464L	COSM22979

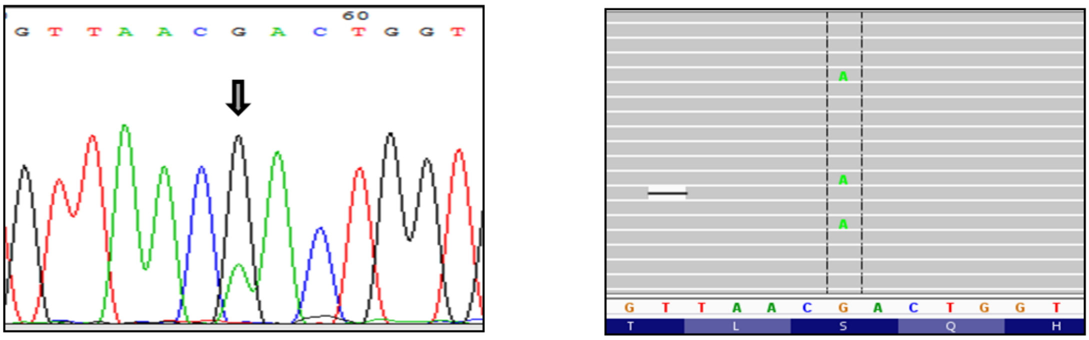

Figure S3. Sanger sequencing validation of *BRAF*, *NRAS*, and *FBXW7* mutations. All mutations found in *BRAF*, *NRAS*, and *FBXW7* were validated with Sanger sequencing. The figure shows one representative sample for each gene. On the left are displayed Sanger chromatograms, while results of NGS, showing read alignment to the reference genome, are on the right of the panel.
